# Biodiversity of cyanobacteria and other aquatic microorganisms across a freshwater to brackish water gradient determined by shotgun metagenomic sequencing analysis in the San Francisco Estuary, USA

**DOI:** 10.1371/journal.pone.0203953

**Published:** 2018-09-24

**Authors:** Tomofumi Kurobe, Peggy W. Lehman, Bruce G. Hammock, Melissa B. Bolotaolo, Sarah Lesmeister, Swee J. Teh

**Affiliations:** 1 Department of Anatomy, Physiology and Cell Biology, School of Veterinary Medicine, University of California, Davis, Davis, California, United States of America; 2 California Department of Fish and Wildlife, Stockton, California, United States of America; 3 California Department of Water Resources, West Sacramento, California, United States of America; Nanjing University, CHINA

## Abstract

Blooms of *Microcystis* and other harmful cyanobacteria can degrade water quality by producing cyanotoxins or other toxic compounds. The goals of this study were (1) to facilitate understanding of community structure for various aquatic microorganisms in brackish water and freshwater regions with emphasis on cyanobacteria, and (2) to test a hypothesis that *Microcystis* genotypes that tolerate higher salinity were blooming in brackish water environments during the severe drought, 2014. Shotgun metagenomic analysis revealed that cyanobacteria dominated the brackish water region while bacteria dominated the freshwater region. A group of cyanobacteria (e.g., *Aphanizomenon*, *Microcystis*, *Planktothrix*, *Pseudanabaena*), bacteria (e.g., *Bacillus*, *Porphyrobacter*), and diatoms (*Phaeodactylum* and *Thalassiosira*) were abundant in the brackish water region. In contrast, *Hassallia* (cyanobacteria) and green algae (*Nannochloropsis*, *Chlamydomonas*, and *Volvox*) were abundant in the landward freshwater region. Station variation was also apparent. One landward sampling station located downstream of an urbanized area differed substantially from the other stations in terms of both water chemistry and community structure, with a higher percentage of arthropods, green algae, and eukaryotes. Screening of the *Microcystis* internal transcribed spacer region revealed six representative genotypes, and two of which were successfully quantified using qPCR (Genotypes I and VI). Both genotypes occurred predominantly in the freshwater region, so the data from this study did not support the hypothesis that salinity tolerant *Microcystis* genotypes bloomed in the brackish water region in 2014.

## Introduction

The San Francisco Estuary (SFE) is the largest estuary on the west coast of North America and provides water for human use and wetland habitat [1]. Over 50% of freshwater inputs originate from the Sierra Nevada, located to the east of the SFE, and this freshwater creates a salinity gradient as it flows into the San Francisco Bay [2]. The hydrodynamics in the SFE is complex because of natural variability (e.g., salinity fluctuations associated with tides and intra- and inter-annual variation in precipitation) as well as various anthropogenic activities (e.g., levee construction, agricultural activities, and freshwater export to Southern California) [2–3]. Nutrients, such as nitrogen and phosphorous, are released from various sources, the largest of which are urbanized areas [4].

Blooms of *Microcystis* spp. have occurred in the SFE during the summer since 1999 [5–6]. *Microcystis* and other harmful cyanobacteria can degrade water quality by producing cyanotoxins or other toxic compounds [7–9]. Laboratory experiments have demonstrated that *Microcystis* directly or indirectly affect the health (e.g., growth) and survival of embryo to sub-adult fishes [10–11]. Coincident with high concentrations of microcystins, Striped Bass (*Morone saxatilis*) collected from the brackish water region of the SFE showed signs of exposure to toxic contaminants or cancer causing substances, including tumor formation, presence of preneoplastic foci, and single cell necrosis in the liver tissue [12]. Blooms of cyanobacteria can also impact the food web of the SFE because cyanobacteria are poor foods for zooplankton (e.g., Copepoda, Cladocera) compared with eukaryotic phytoplankton such as diatoms [13–14]. In addition, *Microcystis* may have a direct negative impact on diatoms and green algae due to allelopathy [15]. By producing toxic compounds as well as impacts on the quality and quantity of food resources at the base of the aquatic food web, *Microcystis* blooms may have contributed to a decline in resident fish species since 2000 [16–18].

California experienced severe drought from 2012–2015. Precipitation was far below average and atmospheric temperature was historically high, resulting in reduced freshwater inflow and high water temperature in major waterways of California [19–20]. In addition, reduced freshwater inflow led to saltwater intrusion into typically freshwater marshes in Franks Tract and the surrounding areas. Thus, the drought may have altered algal assemblages in the SFE by favoring taxa that tolerate higher temperatures and salinity levels. During 2014, intense blooms of *Microcystis* spp. were observed that had the highest biomass, highest toxin concentrations, and the longest duration since *Microcystis* was first documented in 1990 [21]. *Microcystis* blooms occur in freshwater environments, however *Microcystis* can tolerate high salinity such as 7 g L^-1^ of NaCl or even higher [22–24]. Blooming of *Microcystis* in the brackish water region is a potential ecological concern because various fish species, including Delta Smelt *Hypomesus transpacificus*, an endangered fish species in the SFE, occur in the low salinity zone where salinity is 1–6 ppt in summer [25–26]. Therefore, blooms of *Microcystis* in the brackish water region could result in exposure of resident fishes to toxic compounds released from *Microcystis*.

Traditionally, identification of cyanobacteria and phytoplankton was performed by microscopy. Various cyanobacterial genera have been reported in the SFE, including *Aphanizomenon*, *Dolicospermum*, *Oscillatoriales*, *Planktolyngbya*, and *Pseudanabaena* [12, 21, 27]. Morphological identification provides critical information such as accurate taxonomic identification and quantitative data, however, the process is relatively time consuming and requires experienced taxonomists. In addition, taxonomic identification by morphology can sometimes be challenging even for experienced taxonomists due to significant phenotypic changes that may occur in natural assemblages and in laboratory environments [28]. To overcome these issues, molecular techniques such as DNA barcoding, metagenomic analysis, and quantitative PCR (qPCR) are also used for identification and quantification of cyanobacteria and other microorganisms in aquatic environments [29–33].

The goals of this study were (1) to facilitate understanding of community structure for various aquatic microorganisms in brackish water and freshwater regions in the SFE with emphasis on cyanobacteria, and (2) to test a hypothesis that *Microcystis* genotypes that tolerate higher salinity were blooming in brackish water environments during the severe drought, 2014. To achieve these goals we used shotgun metagenomic analysis (hereinafter ‘metagenomic analysis’) on particulate organic matter (POM) collected at six representative sampling stations in the SFE. In addition, we developed quantitative PCR (qPCR) assays for investigating spatiotemporal distribution patterns of *Microcystis* genotypes.

## Materials and methods

### Ethics statement

No specific permissions were required for the sampling stations/activities because the sampling stations were not privately owned or protected. This study did not involve endangered or protected species.

### Site description and sampling stations

**Fig 1:**
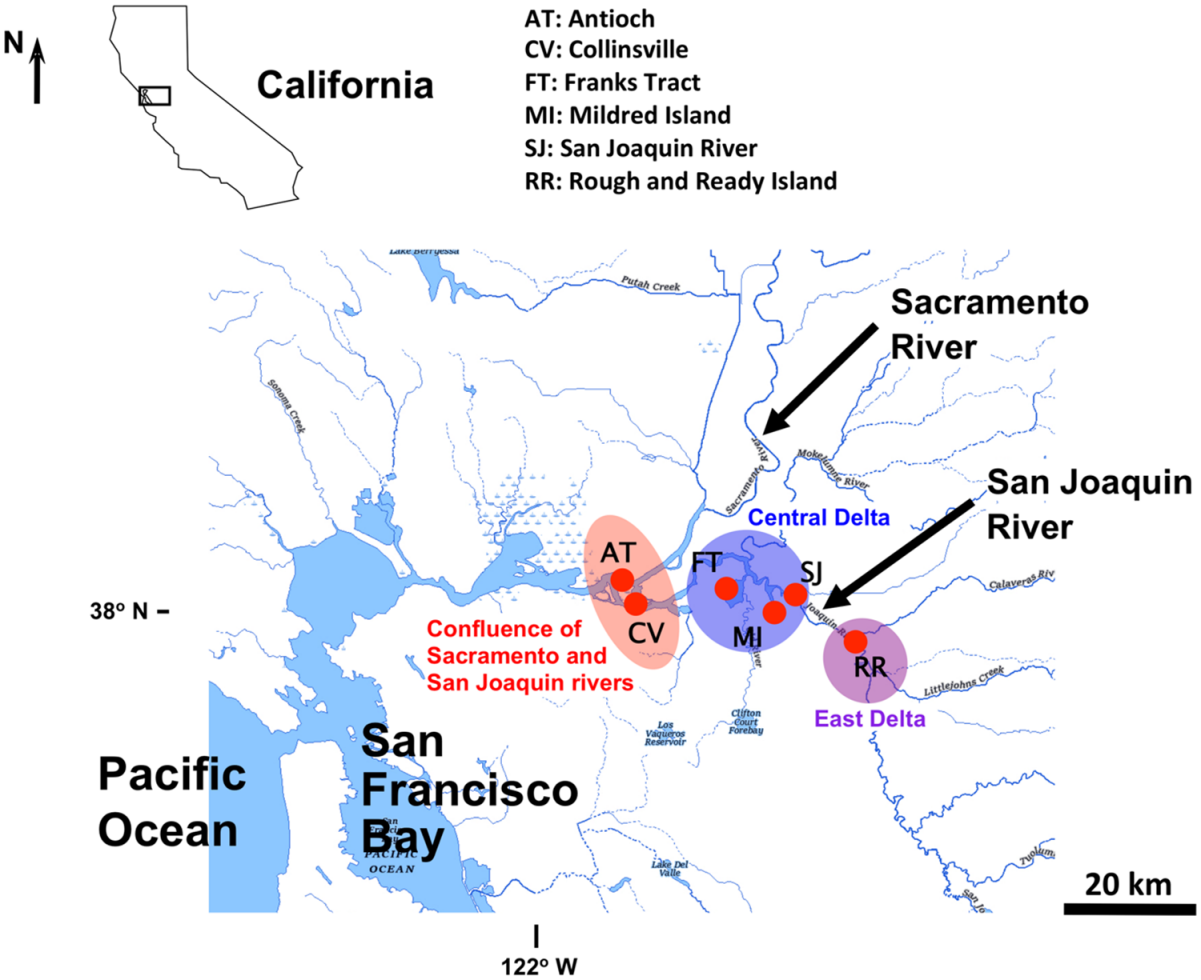
Map of the San Francisco Estuary showing the location of the sampling stations. The base map was downloaded from the U.S. Geological Survey (https://viewer.nationalmap.gov/advanced-viewer/viewer/index.html?extent=-13809717.9302%2C4505236.7823%2C-13369746.3954%2C4697552.3454%2C102100) [35].

The SFE contains 1,100 km of waterways, which receive fresh water from the Sacramento River in the north, the San Joaquin River in the southeast, and marine water from the San Francisco Bay to the west (Fig 1). *Microcystis* blooms occur throughout the summer and fall, however the length of the bloom each year varies with environmental conditions, increasing with water temperature and residence time [21]. *Microcystis* blooms begin in the San Joaquin River and extend both northward and westward into the Sacramento River and Suisun Bay with outflow and tide [5–6]. The main river channels are ~12 m deep and are linked with shallow water habitats in flooded islands and floodplains that are only a few meters deep or less.

The sampling stations for metagenomic analysis and qPCR were located within the bloom region of *Microcystis* in the SFE: Antioch (AT: 38° 1' 23.52'' N, 121° 48' 25.56'' W) and Collinsville (CV: 38° 3' 36.54'' N, 121° 49' 26.33'' W) at the confluence of the Sacramento and San Joaquin rivers; Franks Tract (FT: 38° 2' 48.48'' N, 121° 37' 9.12'' W), Mildred Island (MI: 37° 59' 42.72'' N, 121° 31' 19.92'' W), and San Joaquin River (SJ: 38° 2' 4.92'' N, 121° 29' 0.24'' W) in the central Delta; and Rough and Ready Island (RR: 37° 57' 47.88'' N, 121° 21' 58.68'' W) in the east Delta (Fig 1). At the confluence of the rivers (AT and CV), fresh water from the Sacramento and San Joaquin rivers converge and meet the brackish water seaward, creating an area with salinities ranging from 1–6 [34]. RR is located downstream of the city of Stockton, California. Station FT and MI are flooded islands used for recreation (e.g., fishing, kayaking). Stations FT, MI, and SJ are surrounded by extensive agriculture.

### Sample collection

Field sampling was conducted bi-weekly at the six stations from July-December in 2014. Water temperature, specific conductance (SC), dissolved oxygen (DO) concentration, pH, and turbidity (NTU) were measured at 0.3 m depth using an YSI 6600 sonde (YSI, https://www.ysi.com/). The sonde was calibrated prior to use. Chlorophyll fluorescence was calibrated with a chlorophyll *a* standard (Turner Designs, San Jose, California). SC was converted to salinity [36]. Light attenuation at the surface and at depth within the photic zone was measured by a spherical quantum sensor LI-193 (LI-COR Biosciences, Lincoln, Nebraska) [21]. POM, containing cyanobacteria, phytoplankton, and other organic matter, was collected from the surface of the water column by a gentle hand tow of a 0.3 m diameter plankton net (75 μm mesh) over a distance of 30.5 m. The net was fitted with floats that kept the ring just below the surface, making the net tow an integrated sample of the 0.3 m surface layer [21]. POM collected by tow net was kept on ice, transported to the Aquatic Health Program at the University of California, Davis. POM for metagenomic analysis was harvested by filtration with a nitrocellulose membrane (pore size 0.45 micron, EMD Millipore, Billerica, Massachusetts) using a vacuum filter apparatus, and then stored at −80 °C within 24 h of collection. POM was also used for determination of *Microcystis* biovolume by microscopy (>75 μm size fraction) as well as for measuring chlorophyll *a* concentration by spectrophotometry as described by Lehman et al. [21]. *Microcystis* biovolume and chlorophyll *a* concentration in the net tow were corrected to the total volume of water sampled using a flow meter (Model: 2030R, General Oceanics, Miami, Florida).

In addition to the surface water tow, subsurface ambient water for qPCR as well as for measuring water quality parameters was collected with a van Dorn water sampler at 0.3 m depth. Water for qPCR was filtered with a nitrocellulose membrane (pore size 0.45 micron, EMD Millipore) using a vacuum filter apparatus, and then stored at −80 °C within 24 h of sample collection. Water quality parameters were measured using water filtered through nucleopore filters (0.45 μm pore size) and frozen until analysis of chloride, ammonium, nitrate plus nitrite, silica and soluble reactive phosphorus [37–39]. Water for dissolved organic carbon analysis was filtered through pre-combusted GF/F filters (pore size 0.7 μm) and kept at −20 °C until analysis [39]. Unfiltered water samples for total and volatile suspended solids, total organic carbon, and total phosphate analyses were kept at 4 °C until analysis [39].

### Metagenomic analysis

POM was selected for metagenomic analysis since colony or cluster-forming cyanobacteria (e.g., *Aphanizomenon*, *Cylindrospermum*, *Dolicospermum*, *Microcystis*) were our major interest. POM collected on September 11^th^, 2014, the peak *Microcystis* bloom in 2014, was used for the analysis [21]. One half of the membrane with POM was aseptically cut with scissors and used for genomic DNA (gDNA) extraction using a NucleoSpin Plant II Kit (Macherey-Nagel, Bethlehem, Pennsylvania). The gDNA samples were submitted to the DNA Technology Core Facility at UC Davis (http://dnatech.genomecenter.ucdavis.edu/). The concentrations of gDNA were measured by Qubit fluorometer (ThermoFisher Scientific, Waltham, Massachusetts) and 500 ng of gDNA was used for the library preparation. The gDNA samples were fragmented with a Bioruptor-NGS (Diagenode, Liège, Belgium) during 5 cycles of ultrasound sonication (15 sec. on/ 90 sec. off). The sonicated gDNA samples were concentrated and size-selected using Ampure XP beads (0.6× volume; Beckman Coulter, Brea, California). Libraries for the sequencing reaction were generated with the Kapa Hyper kit (Kapa, Cape Town, South Africa) and barcoded NEXTflex adapters (Bioo-Scientific, Austin, Texas). The libraries were cleaned once more using Ampure XP beads (0.6× volume, Genesee Scientific, San Diego, California), resulting in libraries with an average insert size of approximately 450 bp as measured by BioAnalyzer (Agilent, Santa Clara, California). The concentrations of the library were quantified by Qubit fluorometer, pooled equimolarly, and then sequenced on a MiSeq instrument (Illumina, San Diego, California) with paired-end 250 bp reads with Version 2 chemistry. A total of 28 million reads was obtained by the pair-end sequencing reaction (S1 Table). The DNA sequencing data are available in the NCBI database (BioProject ID: PRJNA434758; BioSample accessions: SAMN08554434-SAMN08554439).

A series of bioinformatics programs were used for the data processing; the sequence data in FASTQ format were subjected to quality check using FastQC ver. 0.11.5, trimming and concatenating forward and reverse reads using PEAR ver. 0.9.6 (overlap length: 10 bp, minimum length: 250 bp) [40–41]. After quality trimming and concatenating pair-end sequences, we obtained a total of 9.2 million reads with an average length of 389 bp for the six libraries (S1 Table). Annotation was performed by running BLASTX sequence similarity searches using DIAMOND ver. 0.7.11 (e-value cutoff of 1 × 10^−10^) [42]. The non-redundant protein database downloaded from the NCBI website on April 11^th^, 2016 (405,117 protein sequences were available at the database http://www.ncbi.nlm.nih.gov/) was used for the BLASTX searches. The output files from the BLASTX analysis were further used for taxonomic analysis using MEGAN ver. 5.11.3 [43–44]. Relative abundance of genera was estimated by counting the number of DNA sequences successfully assigned by BLASTX similarity searches to each node at the genus level, which was further used for investigating taxonomic profiles and generating hierarchical clustering heatmaps. All the data processing was performed using a custom workstation built with 2× Xeon E5-2630 6 core CPU with 256GB ECC RAM, 4× HDD in RAID 10 configuration for Data Storage, 64 bit Linux system with Ubuntu ver. 16.04 LTS.

Hierarchical clustering heatmaps for cyanobacteria, bacteria, and green algae and diatoms were generated at the genus level using the R software package ‘gplots,’ ver. 3.3.3 [45–46]. In this study, organisms belonging to the following phyla were conventionally classified as ‘green algae and diatoms’: Bacillariophyta, Charophyta, Chlorophyta, Cryptophyta, Dinoflagellata, Euglenida, Euglenophyta, Glaucophyta, Haptophyta, and Heterokonta. Organisms belonging to the phylum Cyanobacteria were classified as ‘cyanobacteria’ while the other prokaryotes in the domain ‘Bacteria’ were classified as ‘bacteria’. Rare taxa, defined as a percentage of DNA sequences less than 0.5% of the total in each category, were not included in the heatmaps to conserve space.

### *Microcystis* genotyping

For screening and identification of *Microcystis* genotypes, we utilized the internal transcribed spacer (ITS) region, a conserved region in bacteria and cyanobacteria genomes that is commonly used for species identification and genotyping [47]. DNA sequences encoding the *Microcystis* ITS region were screened by a custom shell script, followed by clustering of DNA sequences using USEARCH ver. 8.1.1861 with a threshold of 99.5% [48]. A portion of the *Microcystis* ITS sequences (approximately 300 bp) was used for creating a multiple alignment by MUSCLE ver. 3.8.31 and the alignment was further used for phylogenetic analysis (S1 Fig) [49]. The DNA sequences used for phylogenetic analysis were deposited in the NCBI database (GenBank accession numbers: MG997093-MG997098). A portion of ITS sequence from *Gloeocapsa* sp. (GenBank accession number: KJ746508.1) was used as an outgroup for generating a rooted phylogenetic tree. A phylogenetic tree was generated by MrBayes program ver. 3.2.1 within Geneious software ver. 6.1.8 with the following settings: Substitution Model: Hasegawa-Kishono-Yano (HKY85) model, Rate Variation: invgamma, Outgroup: *Gloeocapsa* sp. (KJ746508.1), Gamma Categories: 4, Chain Length: 10,000,000, Heated Chains: 4, Heated Chain Temp: 0.5, Subsampling Freq: 200, Burn-in Length: 25,000 [50–51]. The HKY85-invgamma substitution model and rate variation was chosen by jModelTest ver. 2.1.10 as the best model for the dataset [52].

### Quantitative PCR

Quantitative PCR assays for the *Microcystis* genotypes were developed based on the ITS sequences obtained by metagenomic analysis. The probes and primers were manually designed due to the very high similarity of the ITS sequences (S1 Fig). The custom probes labeled with the fluorescent reporter 6-FAM at the 5’ end and with the quencher MGBNFQ at the 3’ end were purchased from ThermoFisher Scientific. Standard curves were generated by running reactions on serial dilutions of plasmid DNA harboring *Microcystis* ITS sequences, which were chemically synthesized and ligated into a cloning vector by a manufacturer (ThermoFisher Scientific). Specificity of the qPCR assays was evaluated by running reactions with all the combinations of the assays and plasmid DNA standards (10–1 × 10^6^ copies per reaction). The qPCR assays for *Microcystis* Genotypes II-V showed cross-reactivity with non-target plasmid DNA standards, therefore these assays were excluded in this study. Thus, only the assays for Genotypes I and IV, for which specificity was successfully validated, were used for analyzing the field samples (S2 Table). Besides *Microcystis* genotypes, abundance of total *Microcystis* (both toxin producing and non-producing, targeting conserved region in the 16S ribosomal RNA gene) and toxin producing *Microcystis* (*mcyD* gene) were measured by qPCR assays [21, 53].

Reactions were performed following our previously reported methods [21, 53]. Briefly, a portion of the membrane (1/4) from subsurface ambient water samples collected by a van Dorn water sampler was used for genomic DNA extraction using NucleoSpin Plant II Kit (Macherey-Nagel). For running reactions, Maxima Probe/ROX qPCR Master Mix was used with addition of BSA at a final concentration of 250 ng μL^−1^ (ThermoFisher Scientific) [54]. The reactions were performed in triplicate using ABI 7900HT Fast Real-Time PCR System available at Real-Time PCR Research and Diagnostics Core Facility at University of California, Davis (http://www.vetmed.ucdavis.edu/vme/taqmanservice/default.html). The qPCR data were visualized using the R packages ‘ggplot2 ver. 2.2.1’ and ‘ggmap ver. 2.6.1’ [55–56]. The qPCR data were square root transformed for graphical comparison [57].

### Statistical analyses

Comparisons of water quality parameters and qPCR data among the sampling stations were performed by ANOVA followed by post hoc Tukey’s HSD tests when statistically significant differences were detected (*p* < 0.05) [46]. Welch's t-test was used to compare the abundances of *Microcystis* Genotype I and VI between the brackish water and freshwater regions [46]. The water quality parameters (dissolved organic carbon, nitrate plus nitrite, salinity, soluble reactive phosphorus, specific conductance, total dissolved solids, total suspended solids) were natural log transformed prior to ANOVAs to meet the assumption of homogeneity of variance. Spearman’s rank correlation coefficients (*r*_*s*_) were calculated to assess the correlation between abundance of *Microcystis* genotypes and water quality parameters by PRIMR-e software ver. 6 (http://www.primer-e.com/) [58–59]. PERMANOVA DISTLM with BEST (Biological-Environmental Stepwise Test) was used to identify the environmental variables that accounted for the most variation in the *Microcystis* abundance data [58–59].

## Results

### Water quality parameters

Water quality parameters varied among the stations (Table 1). Nutrients, such as soluble reactive phosphorous and nitrate plus nitrite, had higher concentrations at RR than the other sampling stations (soluble reactive phosphorous: ANOVA, *F*_[5, 59]_ = 110, *p* < 0.0001; nitrate plus nitrite: ANOVA, *F*_[5, 59]_ = 36, *p* < 0.0001). Dissolved organic carbon concentration was also higher at RR (ANOVA, *F*_[5, 59]_ = 38, *p* < 0.0001). Salinity and other associated environmental variables (specific conductance and chloride concentrations) were higher at the sampling stations at AT and CV than the other landward sampling stations (salinity: ANOVA, *F*_[5, 59]_ = 61, *p* < 0.0001). Similarly, total dissolved solids and total suspended solids were higher at AT and CV then the other stations (total dissolved solids: ANOVA, *F*_[5, 59]_ = 125, *p* < 0.05; total suspended solids: ANOVA, *F*_[5, 59]_ = 45, *p* < 0.0001).

**Table 1:** *Microcystis* biovolume and water quality data for the six sampling stations. Median and median absolute deviation are reported for *Microcystis* biovolume and water quality parameters for samples collected by by-weekly sampling between July and December in 2014, a total of 11 sampling events.

Parameter	Units	AT	CV	FT	MI	SJ	RR
*Microcystis* biovolume (tow)	× 10^9^ μm^3^ L^−1^	0.99 ± 0.99	1.13 ± 1.16	0.51 ± 0.49	1.93 ± 1.70	0.95 ± 0.74	1.66 ± 1.65
Water Temperature	°C	20.7 ± 2.0	20.3 ± 1.6	21.8 ± 1.9	22.4 ± 2.8	22.2 ± 3.0	24.9 ± 1.8
Dissolved Oxygen	mg L^−1^	8.1 ± 0.2	8.2 ± 0.1	9.0 ± 0.5	8.5 ± 0.3	7.6 ± 0.3	7.7 ± 0.6
Percent Dissolved Oxygen	%	91.8 ± 2.0	92.5 ± 3.0	103.0 ± 12.4	98.1 ± 7.0	85.6 ± 6.4	90.8 ± 8.15
Specific conductance	μS cm^−1^	5404 ± 1478	7388 ± 1405	909 ± 141	471 ± 45	393 ± 37	1340 ± 195
Salinity		2.9 ± 0.9	4.1 ± 0.8	0.4 ± 0.1	0.2 ± 0.0	0.2 ± 0.0	0.7 ± 0.1
Chloride	mg L^−1^	1492 ± 372	2178 ± 510	206 ± 33	84 ± 9	61 ± 4	285 ± 55
Turbidity	NTU	13.1 ± 3.8	14.3 ± 4.2	4.0 ± 1.0	0 ± 0.6	1.4 ± 0.6	2.8 ± 0.9
pH		7.8 ± 0.2	7.7 ± 0.1	8.2 ± 0.4	8.2 ± 0.2	7.8 ± 0.3	7.8 ± 0.2
Ammonium	mg L^−1^	0.05 ± 0.01	0.03 ± 0.01	0.03 ± 0.01	0.02 ± 0.00	0.02 ± 0.01	0.08 ± 0.04
Nitrate plus nitrite	mg L^−1^	0.34 ± 0.04	0.39 ± 0.06	0.18 ± 0.06	0.13 ± 0.07	0.18 ± 0.06	1.74 ± 0.47
Total Phosphorus	mg L^−1^	0.11 ± 0.01	0.12 ± 0.01	0.10 ± 0.01	0.10 ± 0.01	0.12 ± 0.01	0.50 ± 0.09
Soluble Reactive Phosphorus	mg L^−1^	0.09 ± 0.01	0.09 ± 0.01	0.08 ± 0.01	0.08 ± 0.01	0.08 ± 0.01	0.42 ± 0.09
Dissolved Silicate	mg L^−1^	13.9 ± 0.5	14.0 ± 0.3	14.4 ± 2.2	15.9 ± 1.2	15.7 ± 1.2	13.5 ± 0.8
Total Dissolved Solids	mg L^−1^	3020 ± 776	4372 ± 1010	521 ± 103	266 ± 26	220 ± 23	740 ± 115
Total Suspended Solids	mg L^−1^	16.0 ± 4.0	19.0 ± 5.0	2.0 ± 0	2.0 ± 0	4.0 ± 1.0	6.0 ± 1.0
Volatile Suspended Solids	mg L^−1^	3.0 ± 1.0	4.0 ± 1.0	1.0 ± 0	1.0 ± 0.0	1.0 ± 0.0	2.0 ± 0.0
Chlorophyll *a* (tow)	μg L^−1^	0.61 ± 0.46	0.28 ± 0.22	1.06 ± 0.39	1.25 ± 1.08	1.13 ± 0.93	1.72 ± 1.57
Chlorophyll *a* (field)	μg L^−1^	1.8 ± 0.4	2.1 ± 0.3	1.8 ± 0.6	2.8 ± 0.3	2.4 ± 0.2	3.3 ± 0.6
Phaeophytin	μg L^−1^	0.019 ± 0.009	0.023 ± 0.005	0.033 ± 0.022	0.038 ± 0.025	0.056 ± 0.040	0.026 ± 0.020
Dissolved Organic Carbon	mg L^−1^	2.3 ± 0.2	2.2 ± 0.3	2.6 ± 0.1	3.2 ± 0.1	3.3 ± 0.3	4.7 ± 0.4
Total Organic Carbon	mg L^−1^	2.5 ± 0.2	2.3 ± 0.2	2.7 ± 0.1	3.3 ± 0.2	3.4 ± 0.2	4.8 ± 0.6
Dissolved Organic Nitrogen	mg L^-1^	0.4 ± 0.1	0.3 ± 0.1	0.2 ± 0.0	0.3 ± 0.1	0.3 ± 0.0	0.6 ± 0.1
Integrated Euphotic Zone Light	μmole m^-1^ s^-1^	814 ± 78	851 ± 96	1445 ± 599	1048 ± 210	973 ± 176	850 ± 244

### Taxonomic profiles

**Fig 2:**
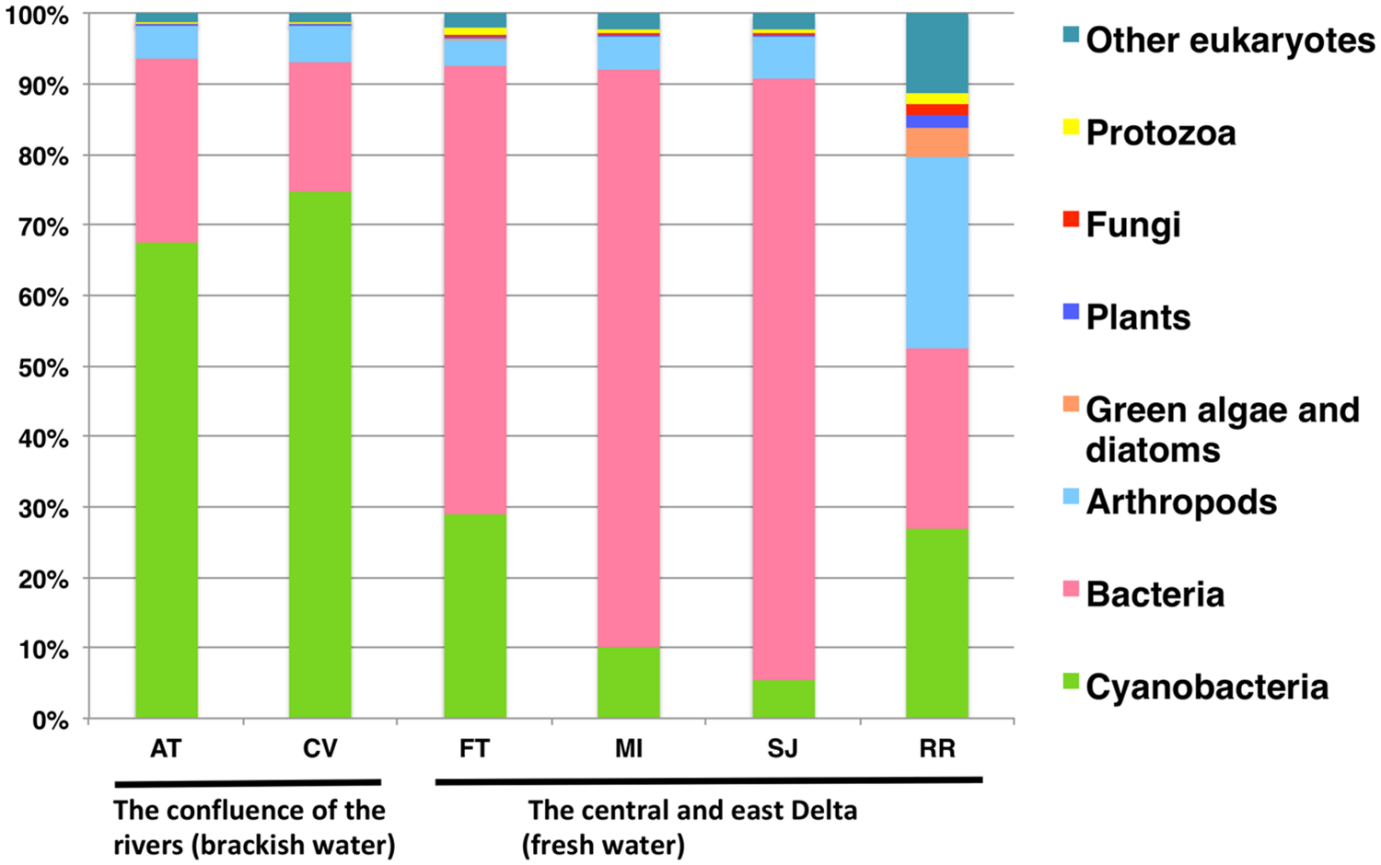
Relative abundance of microscopic aquatic taxa determined by metagenomic analysis using particulate organic matter (POM) collected on September 11^th^, 2014. POM collected by phytoplankton tow net was used for metagenomic analysis, therefore microorganisms smaller than the mesh size (75 μm) are underestimated.

Metagenomic analysis revealed a contrast in the taxonomic community structure between brackish region (AT and CV) and the inland freshwater region (FT, MI, SJ, and RR) (Fig 2). Cyanobacteria were dominant at the two brackish stations whereas bacteria were dominant at the inland stations FT, MI, and SJ (Fig 2). The taxonomic profile of station RR differed from the other sampling stations and was characterized by a higher percentage of arthropods (e.g., *Daphnia* and rotifers), green algae (e.g., *Volvox* and *Chlamydomonas*), and other eukaryotic microorganisms (e.g., fungi, protozoa) than the other stations (Fig 2).

### Cyanobacteria

**Fig 3:**
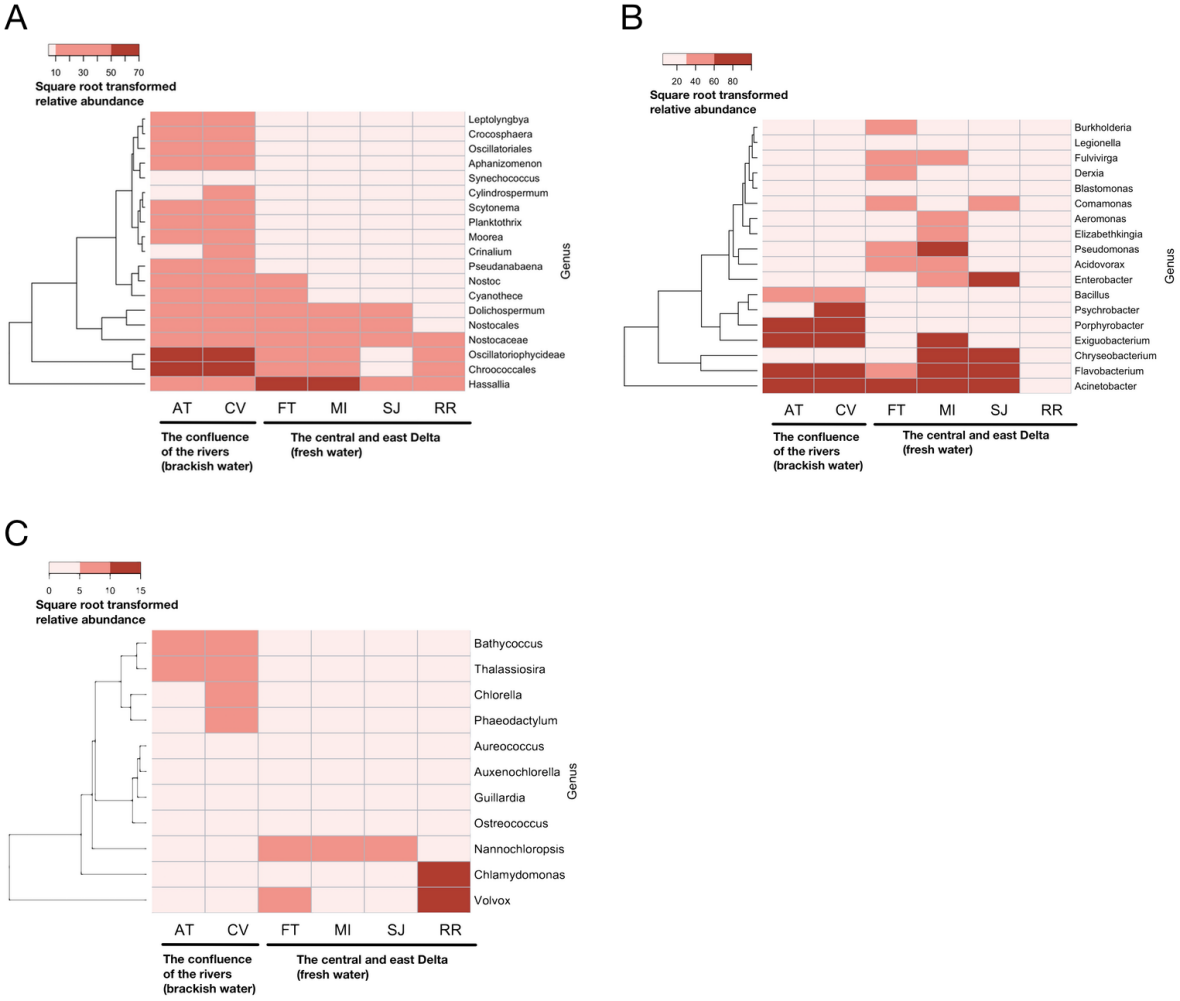
Relative abundance of cyanobacteria (A), bacteria (B), and green algae and diatoms (C) on September 11^th^, 2014 depicted by hierarchical clustering heatmaps. Relative abundance was estimated by counting the number of DNA sequences successfully assigned by BLASTX similarity search to each node at genus level. Rare taxa (relative abundance <0.5%) were not included in the figures. *Microcystis* was excluded from the figure due to its overwhelming abundance in the dataset.

*Microcystis* was found to be the most abundant genus of cyanobacteria, accounting for 94.4% of the cyanobacteria population at the six sampling stations. Besides *Microcystis*, over 19 cyanobacteria genera were detected from at least one of the sampling stations by metagenomic analysis (Fig 3A). Some of the cyanobacteria included species that produce cyanotoxin(s), such as *Aphanizomenon*, *Cylindrospermum*, *Leptolyngbya*, *Moorea*, and *Planktothrix*. Among the cyanobacteria detected, the relative abundances of nine cyanobacteria (*Aphanizomenon*, *Croococcales*, *Crocosphaera*, *Leptolyngbya*, *Moorea*, *Pseudanabaena*, *Planktothrix*, *Oscillatoriales*, *and Scytonema*) were higher at the brackish water (AT and CV) than the inland freshwater stations. In contrast, the cyanobacterium, *Hassalia*, was predominantly found at FT and MI. A large number of DNA sequences encoding microcystin synthetase genes were found (a total of 6,597 DNA sequences from the six sampling stations). However, other cyanotoxin-synthetase genes such as anatoxin and saxitoxin synthetase genes were not detected by metagenomic analysis.

### Bacteria

The most common taxa were environmental bacteria, (e.g., *Acinetobacter*, *Chryseobacterium*, *Exiguobacterium*, *Flavobacterium*, *Porphyrobacter*) (Fig 3B). In addition, coliforms (e.g., *Enterobacter*) and genera including pathogenic species were also detected (e.g., *Aeromonas*, *Legionella*, *Pseudomonas*) (Fig 3B). The relative abundance of bacteria differed between the brackish water and freshwater regions. *Bacillus*, *Porphyrobacter*, and *Psychrobacter* were abundant in at least one of the sampling stations in brackish water, while *Acidovorax*, *Comamonas*, *Chryseobacterium*, *Enterobacter*, *Fulvivirga*, and *Pseudomonas* were abundant at two of the freshwater sampling stations (Fig 3B). *Acinetobacter* and *Flavobacterium* were detected at both brackish and freshwater stations (Fig 3B).

### Green algae and diatoms

A total of 11 green algae and diatoms were detected among the six stations by metagenomic analysis, including *Chlamydomonas*, *Nannochloropsis*, and *Volvox* (green algae), and *Phaeodactylum* and *Thalassiosira* (diatoms) as shown in Fig 3C. The green algae, *Volvox* and *Chlamydomonas*, were mostly found at the freshwater station RR while the diatoms, *Phaeodactylum* and *Thalassiosira*, were detected at the brackish water stations (AT and CV).

### Abundance of total and toxin producing *Microcystis* quantified by qPCR

**Fig 4:**
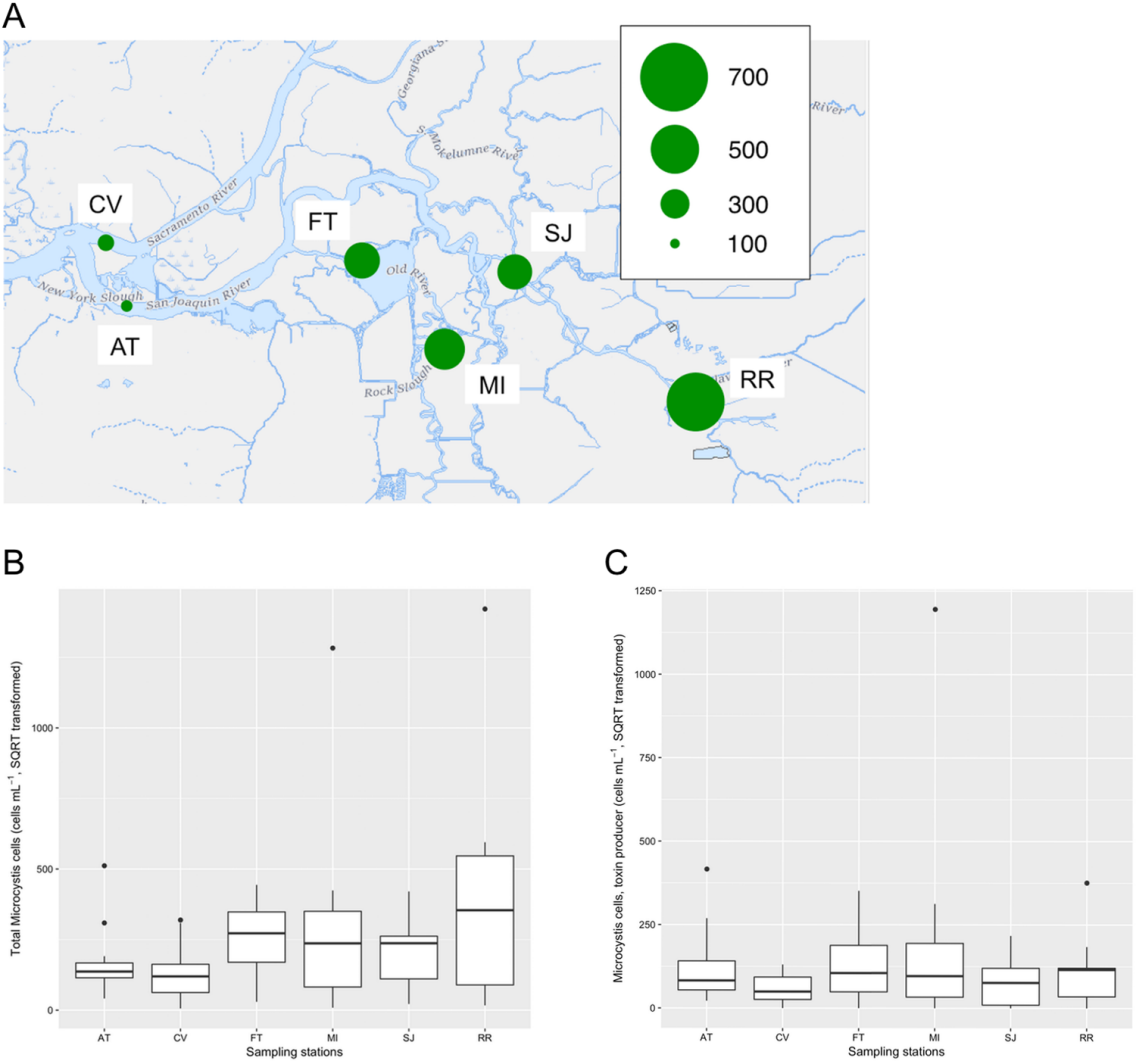
Abundance of total *Microcystis* (cells per mL, square root transformed) on September 11^th^, 2014 (A), among the sampling stations (July-December 2014) (B), and toxin producing *Microcystis* (C) measured by qPCR using subsurface ambient water samples. The base map for panel A was downloaded from the U.S. Geological Survey (https://viewer.nationalmap.gov/advanced-viewer/viewer/index.html?extent=-13600395.1282%2C4561570.8721%2C-13494224.0959%2C4607968.1483%2C102100) [35].

Total *Microcystis* abundance was higher at the freshwater stations (FT, MI, SJ, and RR) than the brackish stations (AT and CV) on September 11^th^, 2014 (Fig 4A). Based on data from the entire survey (July-December 2014), the highest total *Microcystis* abundance was observed at RR, although the difference was not statistically significant (ANOVA, *F*_[5, 59]_ = 1.5, *p* = 0.213) (Fig 4B). Similarly, the abundance of toxin producing *Microcystis* was higher at the freshwater sampling stations, FT, MI, and RR, than the ones at the brackish water stations throughout the survey, however the differences among stations were not statistically significant (ANOVA, *F*_[5, 59]_ = 1.1, *p* = 0.403) (Fig 4C).

### *Microcystis* genotypes

**Fig 5:**
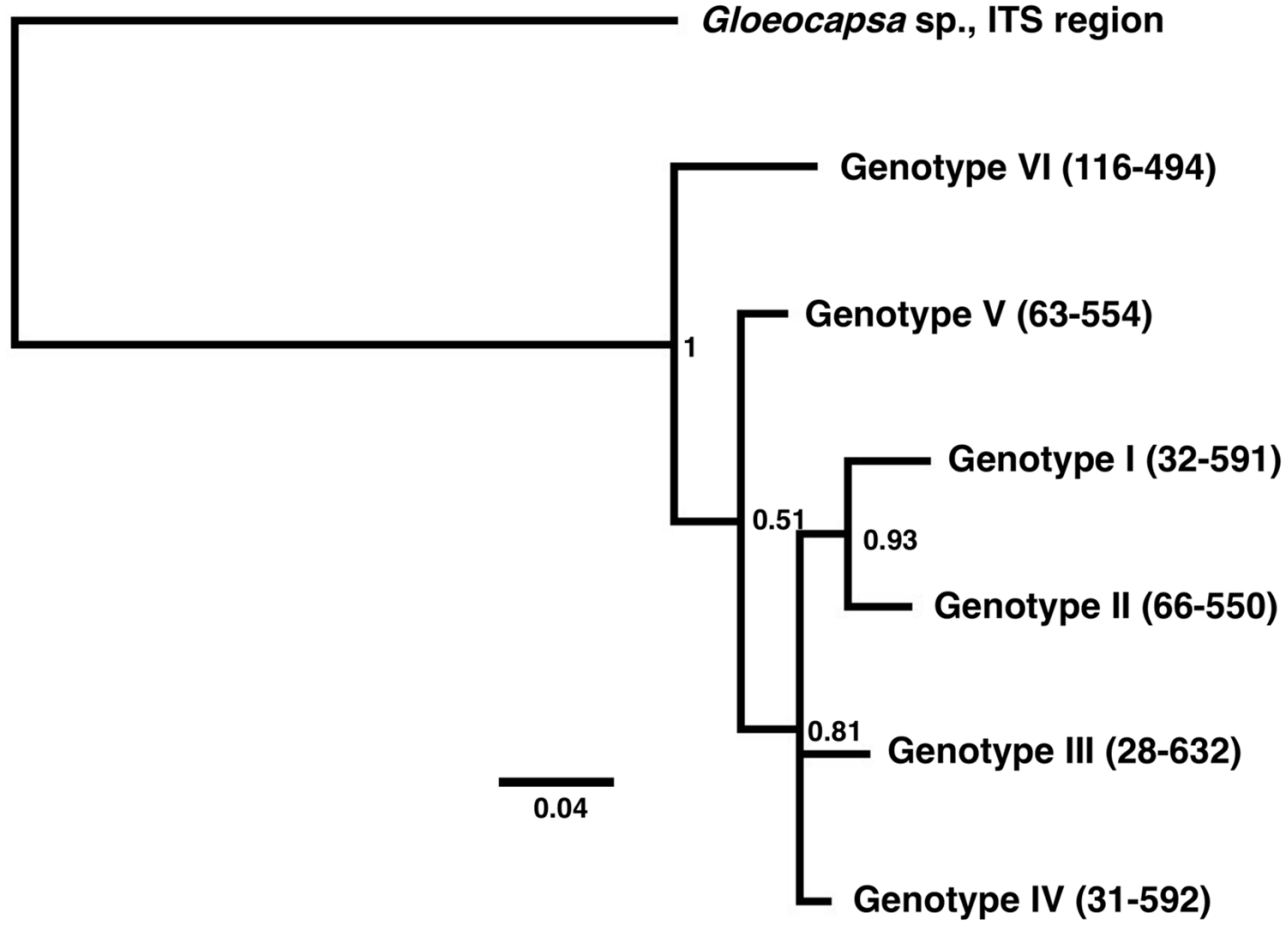
Phylogenetic tree for the six *Microcystis* genotypes generated based on the internal transcribed spacer (ITS) region. The number at each node represents the posterior probability value. The scale bar indicates inferred nucleotide substitution rate. A portion of ITS sequence from *Gloeocapsa* sp. (GenBank accession number: KJ746508.1) was used as an outgroup for generating the rooted phylogenetic tree. The numbers in the parentheses are the internal reference ID number.

A total of 196 DNA sequences encoding the *Microcystis* ITS region were obtained by screening the data from metagenomic analysis. The number was significantly reduced by clustering, resulting in six representative sequences. These representative *Microcystis* ITS sequences were tentatively designated as *Microcystis* Genotype I-VI. The pairwise sequence similarities of the *Microcystis* ITS regions for the six genotypes ranged from 89.2% (the lowest similarity observed between Genotype I and VI) to 95.5% (the highest between Genotype IV and both Genotype I and II) as shown in Table 2. The phylogenetic tree indicates that Genotypes I-V were closely related to each other, and differed substantially from Genotype VI (Fig 5). The *Microcystis* ITS region for Genotype I was identical to that for *Microcystis* spp. reported from Erhai Lake in China (GenBank accession numbers: KF207342.1 and KF207087.1), Joumine Reservoir in Tunisia (HQ389359.1 and HQ389360.1), and Loire River in France (FJ474921.1) [60–62]. *Microcystis* Genotype VI had an identical ITS sequence to *Microcystis* spp. from Lake Taihu and Qinhuai River in China (HQ625397.1 and HQ713834.1) [63–64].

**Table 2:** Pairwise comparisons of DNA sequence similarities for a portion of the internal transcribed spacer (ITS) region obtained from the six *Microcystis* genotypes and *Gloeocapsa* sp. The numbers in the table indicate percentages of DNA sequence similarities.

*Microcystis* genotype	I	II	III	IV	V	VI	*Gloeocapsa* sp.
I	100						
II	94.8	100					
III	93.7	94.3	100				
IV	95.5	95.5	94.4	100			
V	93.0	94.3	94.0	94.8	100		
VI	89.2	89.4	91.2	90.3	93.0	100	
*Gloeocapsa* sp.	63.5	63.5	63.2	64.8	64.2	63.2	100

### Spatiotemporal distribution pattern of *Microcystis* genotypes

**Fig 6:**
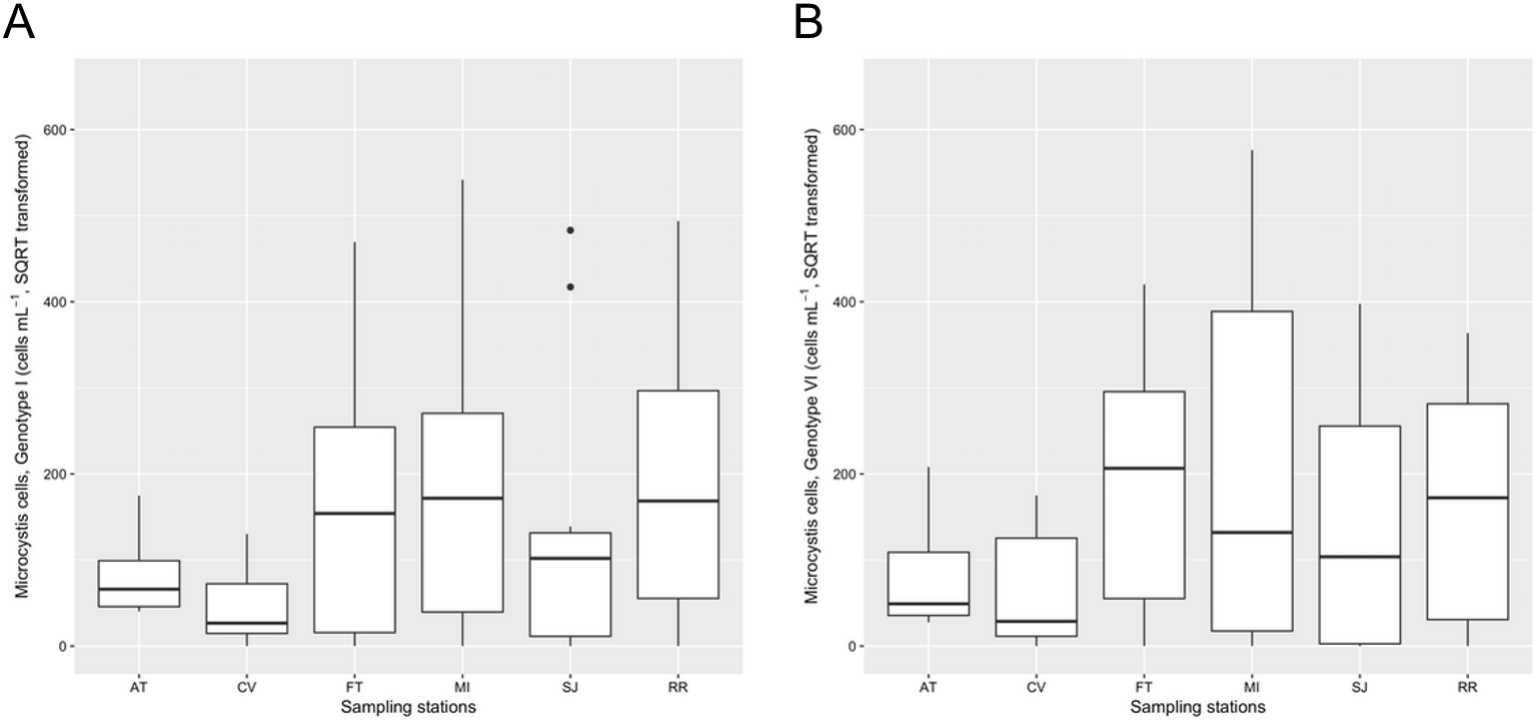
Abundance of *Microcystis* Genotype I (A) and VI (B) among sampling stations between July and December 2014 measured by qPCR (square root transformed) using subsurface ambient water samples.

**Fig 7:**
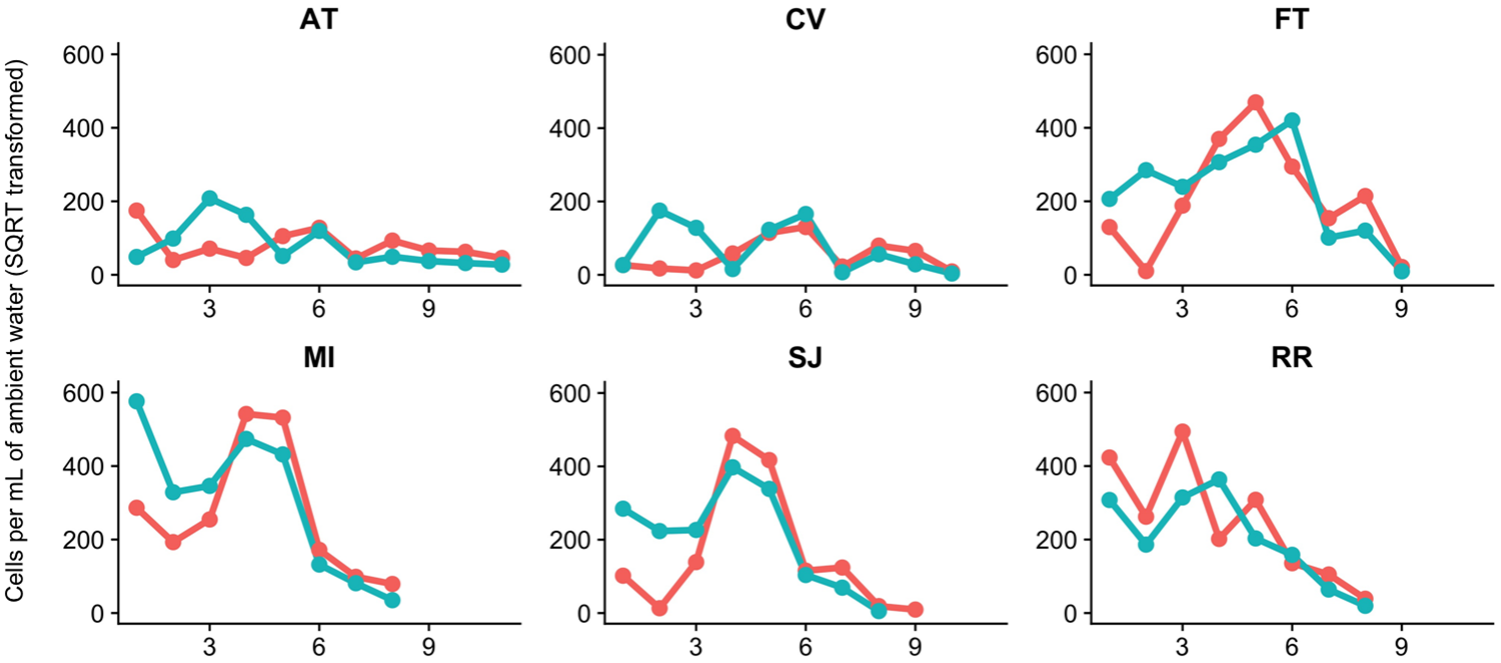
Temporal dynamic changes of *Microcystis* Genotype I (red) and VI abundance (teal) at the six sampling stations measured by qPCR (square root transformed) using subsurface ambient water samples. The x-axis indicates survey identification numbers (1: July 14^th^; 2: July 28^th^; 3: August 11^th^; 4: August 25^th^; 5: September 11^th^; 6: September 29^th^; 7: October 13^th^; 8: October 27^th^; 9: November 10^th^; 10: November 24^th^; 11: December 10^th^, 2014).

*Microcystis* Genotypes I and VI showed a very similar spatiotemporal distribution pattern and were highly correlated (*r*_*s*_ = 0.76; *p* < 0.05) (Figs 6 and 7). There was no clear trend observed regarding the salinity distribution of the two *Microcystis* genotypes (S2 Fig). The abundances of both genotypes were significantly lower at the brackish water than the freshwater region (Genotype I: Welch’s *t*-test, *t*(43) = −0.40, *p* < 0.001; Genotype VI: Welch’s *t*-test, *t*(46) = −0.42, *p* < 0.001) (Fig 6A and 6B). Individual correlations suggested that *Microcystis* Genotype VI had a slightly stronger negative correlation with chloride concentration (*r*_*s*_ = −0.27) than Genotype I (*r*_*s*_ = −0.22). In addition, *Microcystis* Geneotype VI was more positively correlated with water temperature (*r*_*s*_ = 0.55) than Genotype I (*r*_*s*_ = 0.41). Both *Microcystis* Geneotype I (*r*_*s*_ = −0.20) and Genotype VI (*r*_*s*_ = −0.23) were similarly correlated with total suspended solids. In addition, *Microcystis* Genotype VI was more closely correlated with toxic *Microcystis* (*r*_*s*_ = 0.50) than *Microcystis* Genotype I (*r*_*s*_ = 0.20). The PERMANOVA DISTLM with BEST analysis determined that ambient water temperature described most of the variation in the two genotypes (positive correlation) with euphotic zone light, specific conductance, and dissolved organic nitrogen adding to the variance described (Table 3).

**Table 3:** Environmental variables that described the largest percentage of the variation in the abundance of *Microcystis* Genotype I and VI based on PERMANOVA DISTLM with BEST analysis.

	Number of variables	Cumulative adjusted R^2^	Significance (*p*)	Variable
Genotype I	1	0.49	0.001	WT
	2	0.53	0.001	LITE
Genotype VI	1	0.60	0.001	WT
	2	0.67	0.01	SC
	3	0.69	0.02	DON

Abbreviations: DON: dissolved organic nitrogen; LITE: euphotic zone light; SC: specific conductance; WT: water temperature

There were slight differences in the timing of the occurrence of the two *Microcystis* genotypes. The abundance of Genotype VI was slightly higher than that of Genotype I at FT, MI, and SJ on July 14^th^ and 28^th^, 2014 (Fig 7). Similarly, the abundance of Genotype VI was higher at AT and CV on August 11^th^ and August 25^th^, 2014.

## Discussion

Metagenomic analysis revealed that *Microcystis* was the most dominant genus among the cyanobacteria in the surface water during the 2014 drought in freshwater and brackish water habitats. This finding is congruent with the data obtained by microscopy showing *Microcystis* comprised over 80% of the total cyanobacteria and phytoplankton biovolume in the subsurface water in early September 2014 [21, 65]. Although other cyanobacteria that possibly produce saxitoxin, anatoxin-a, and cylindrospermopsin were detected by the metagenomic analysis, the DNA sequences encoding these cyanotoxin synthetase genes (e.g., *sax*, *ana*, and *cyr*) were not found in the DNA sequencing data. This is likely due to either the overwhelming abundance of *Microcystis* in the samples and/or low abundance of cyanobacteria with cyanotoxin synthetase gene. Additional screening by degenerate PCR was able to amplify DNA fragments for *anaF* and *saxA*, which are involved in production of anatoxin and saxitoxin, indicating anatoxin and saxitoxin producing cyanobacteria were also present in the SFE during the severe drought (GenBank accession numbers: MF959540 and MF959541).

The detection of six *Microcystis* genotypes suggests that there are multiple *Microcystis* species in the SFE. The pairwise DNA sequence similarities of the ITS region for the six *Microcystis* genotypes are below the cutoff value commonly used for species identification in bacteria (97.0%) (Table 2) [46]. Particularly *Microcystis* Genotype VI is likely distinct from the other *Microcystis* genotypes found in this study since Genotype VI shows a pairwise DNA sequence similarity below 93% to the other genotypes (Table 2). The ITS region is widely used for species identification, as well as for investigating geographical associations for cyanobacteria and other organisms, including bacteria, fungi, and plants, because the ITS region is highly conserved within species yet has a higher degree of variation than other genes commonly used for identification [66–68]. The substitution rate of DNA sequence in the ITS region and its adjacent region, 16S rRNA gene, is estimated as 1.8 × 10^−3^ per site per million years in bacteria [69]. Interestingly, the ITS sequences for *Microcystis* Genotypes I and VI in the SFE were identical to that of *Microcystis* isolated or detected in other geographic locations, such as Tunisia and France (Genotype I) and China (Genotype I and VI), suggesting possible exchange of *Microcystis* between the SFE and other locations worldwide. Although speculative, the *Microcystis* genotypes could be transported from various locations through international trading (e.g., ballast water) [1]. In any case, our results suggest the presence of multiple *Microcystis* species. To date, three *Microcystis* species have been identified microscopically in SFE, including *M*. *aeruginosa*, *M*. *flos-aquae*, and *M*. *wesenbergii* [21].

*Microcystis* genotypes may occupy slightly different ecological niches. The spatiotemporal distribution of *Microcystis* Genotype I and VI were very similar, however the bloom of *Microcystis* Genotype VI occurred slightly earlier than that of Genotype I (Figs 6 and 7). Water temperature could be an especially important factor affecting the abundance of Genotype VI, because Genotype VI was more closely correlated with water temperature than Genotype I. Moisander et al. [34] demonstrated the presence of multiple *Microcystis* genotypes based on the *mcyA* and *cpcBA* genes and discussed the potential for salinity tolerant strains or genotypes of *Microcystis* in the SFE. We hypothesized presence of salinity tolerant *Microcystis* genotypes and their blooms in the brackish water region during the severe drought, however our results did not support this hypothesis. Both *Microcystis* Genotypes I and VI occurred predominantly in the freshwater region. Further analysis is needed to identify the presence of salinity tolerant *Microcystis* genotypes in the SFE. Recently, Otten et al. [33] identified eight *Microcystis* operational taxonomic units (OTUs; synonymous with individual strains) using algal samples collected in 2011 and 2012 in the SFE and investigated their spatiotemporal distribution patterns in the brackish water and freshwater regions. Similar to our findings, the researchers did not find evidence for site-specific, endemic *Microcystis* populations [33].

Various types of bacteria were detected by the metagenomic analysis, such as environmental bacteria (e.g., *Acinetobacter* and *Flavobacterium*), coliforms (*Enterobacter*), and two pathogenic species (*Aeromonas*, *Legionella*). These bacteria might have used dissolved organic matter from *Microcystis* or other cyanobacteria for their growth. One of the major ecological roles of bacteria is scavenging of the major biologically-important elements: carbon, nitrogen and phosphorus. Particularly, carbon (DOC) is often the primary substrate for bacterial growth in aquatic ecosystems and DOC released from phytoplankton and cyanobacteria is thought to be high quality carbon for bacterial growth [70–71]. In this study, we found that the relative abundance of *Aeromonas* and *Enterobacter* was high at inland freshwater sampling stations in which *Microcystis* was abundant. *Aeromonas* are ubiquitously present in aquatic ecosystems and fish culture facilities, and cause diseases in fishes when the abundance of the bacteria is enhanced by abiotic factors (e.g., organic pollutants, water temperature) or when fish immune system is compromised by stressors (e.g., inadequate water quality, low dissolved oxygen) [72–73]. Recent research demonstrated that *Aeromonas* growth was enhanced by *Microcystis* lysate [10], which may contribute to the increase in *Aeromonas* in inland water. *Enterobacter* belong to the coliform group and can be found widely in nature through excretion from the intestinal tracts of animals [74]. Several species in the genus are pathogenic and cause opportunistic infections such as eye and skin infections, meningitis, bacteremia (bacterial blood infection), pneumonia, and urinary tract infections in immunologically compromised individuals [74]. Blooms of *Microcystis* as well as high concentrations of microcystins were major health concerns during the severe drought in the SFE because microcystins cause hepatic toxicity in wild and domestic animals, fishes, and humans [8–10]. In addition, proliferation of pathogenic bacteria on dissolved organic matter from *Microcystis* and other cyanobacteria could further degrade water quality, posing an additional public health risk.

The highest percentage of microscopic aquatic eukaryotes (e.g., *Daphnia*, copepods, protozoa) was observed at RR, located downstream of the city of Stockton. The high phosphorous and nitrogen at RR may have contributed to the higher percentage of the green algae *Volvox* and *Chlamydomonas* and the accompanying abundance of primary consumers at the station. Cieminski and Flake [75] reported that nitrogen and phosphorus from municipalities generally enhance the growth of phytoplankton that can further support zooplankton populations. *Chlamydomonas* in particular may be beneficial to *Daphnia* since *Chlamydomonas* was found to be a major prey item of *Daphnia* by electron microscopy [76]. The water quality at RR seems to be suitable for green algae as well as microscopic aquatic eukaryotes, however a high abundance of *Microcystis* was also found at the station (Fig 4A), suggesting that *Microcystis* may use nutrients released from the municipality as well. In addition, it is noteworthy that the abundance of *Microcystis* at RR was numerically higher than the other stations while relative abundance of *Microcystis* at the same station was lower due to the higher abundance of other aquatic microorganisms (Figs 2 and 4A). Thus, while metagenomic analysis is a powerful tool to investigate community structure of microorganisms, quantification of organisms of interest by additional endpoints such as qPCR can provide more meaningful data to understand population dynamics.

## Conclusion

In this study, we investigated biodiversity of cyanobacteria and other aquatic microorganisms in the SFE using metagenomic analysis during a *Microcystis* bloom in 2014. Our data indicate that aquatic microorganisms formed unique regional population structures. Cyanobacteria dominated the brackish water region, bacteria dominated the inland freshwater region, and green algae as well as other microscopic eukaryotes were observed in the freshwater station downstream of the urbanized area. Metagenomic analysis also revealed the presence of multiple *Microcystis* genotypes, which likely belong to different species in the genus. Salinity tolerance of the *Microcystis* genotypes and their ecological niche in the SFE are still unknown.

As demonstrated in this study, metagenomic analysis coupled with qPCR is a powerful approach for investigating biodiversity and spatiotemporal dynamic changes of microscopic organisms, however the approach does not always provide conclusive results. For example, co-occurrence of green algae (*Volvox* and *Chlamydomonas*) and primary consumers (*Daphnia*, copepods, and other protozoa) at RR could be a coincident event. Therefore, performing interdisciplinary studies encompassing various endpoints (e.g., molecular analyses, morphological identification, isotope analyses, water quality measurement), followed by laboratory experiments using live organisms, is essential to better understand the complex interactions of aquatic microorganisms.

## Supporting information

S1 Fig Multiple alignment of ITS regions for the six *Microcystis* genotypes. The numbers in the parentheses indicate internal sequence identification.
(TIF)

S2 Fig Scatter plot between salinity and two *Microcystis* genotypes, I and VI. The abundance of *Microcystis* genotypes was quantified by qPCR using subsurface ambient water.
(TIF)

S1 Table Summary for the sequencing output and annotation for the six sampling stations. (TIFF)

S2 Table Probe and primer sequences for qPCR assays used in this study. (TIFF)
