# Supplementary figures and images for: Biodiversity of cyanobacteria and other aquatic microorganisms across a freshwater to brackish water gradient determined by shotgun metagenomic sequencing analysis in the San Francisco Estuary, USA
Source: PLoS One. 2018 Sep 24;13(9):e0203953. doi: 10.1371/journal.pone.0203953 (PMC6152961; doi:10.1371/journal.pone.0203953)

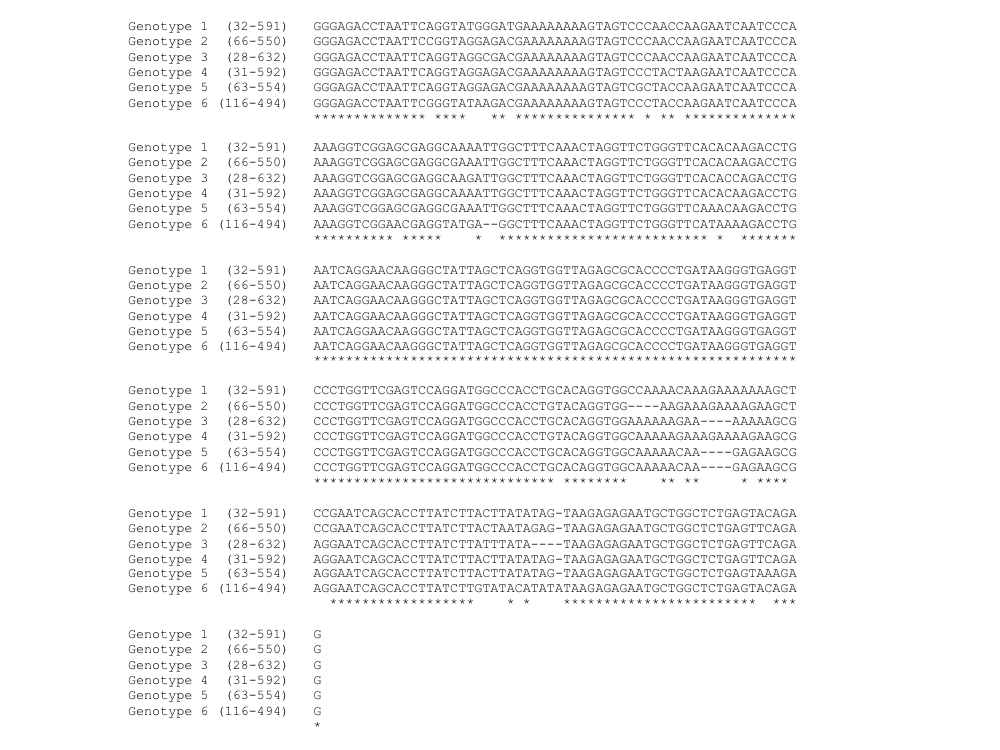

Supplement: S1 Fig — The numbers in the parentheses indicate internal sequence identification. (TIF) [file pone.0203953.s001.tif]

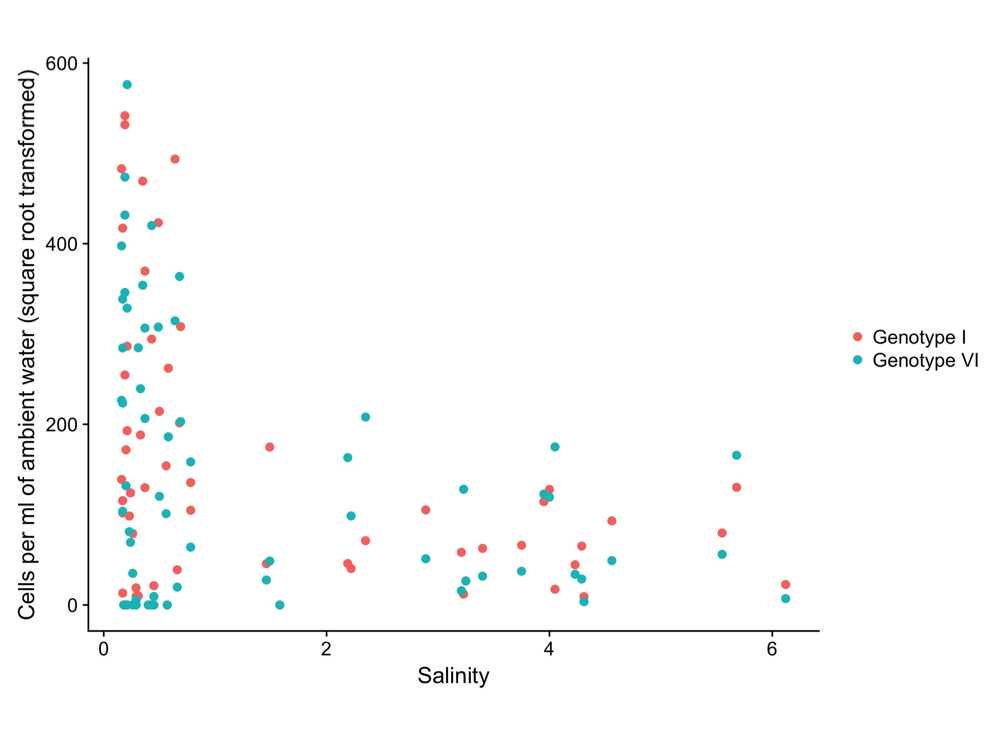

Supplement: S2 Fig — The abundance of Microcystis genotypes was quantified by qPCR using subsurface ambient water. (TIF) [file pone.0203953.s002.tif]

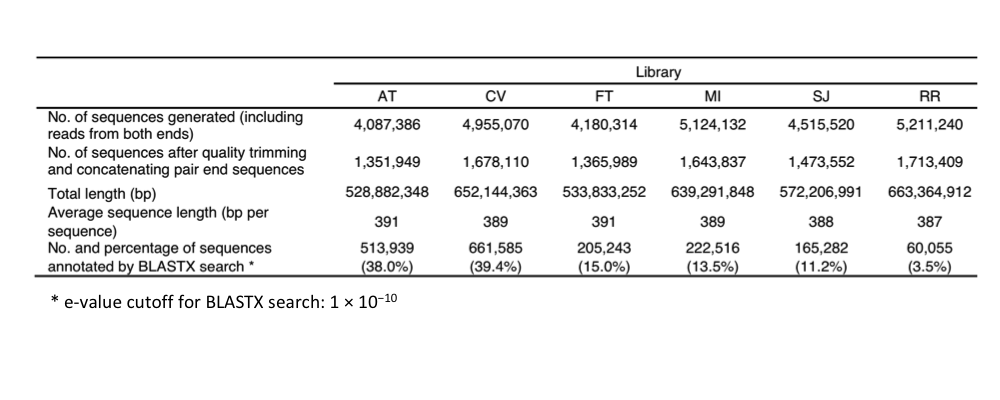

Supplement: S1 Table — (TIFF) [file pone.0203953.s003.tiff]

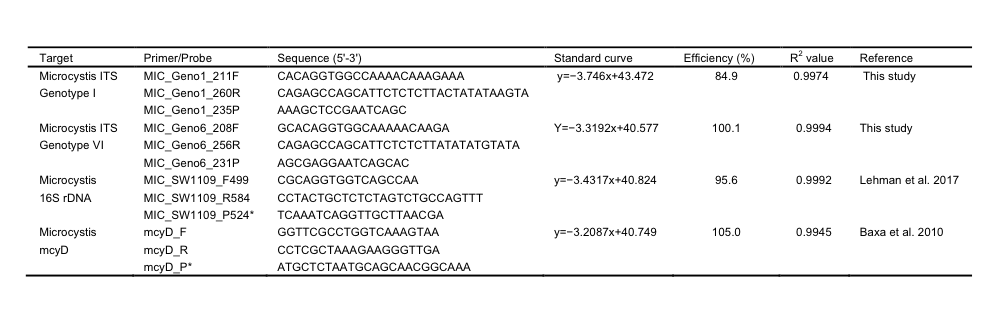

Supplement: S2 Table — (TIFF) [file pone.0203953.s004.tiff]
